# Power effects on interindividual and intergroup competition

**DOI:** 10.1111/bjso.12831

**Published:** 2025-03-17

**Authors:** Tim Wildschut, Chester A. Insko

**Affiliations:** ^1^ School of Psychology University of Southampton Southampton UK; ^2^ Department of Psychology and Neuroscience University of North Carolina Chapel Hill USA

**Keywords:** competition, discontinuity effect, fate control, intergroup, personal power, power, reflexive control, social power

## Abstract

Interindividual‐intergroup discontinuity refers to the finding that groups are more competitive than individuals. Research on this phenomenon has typically compared interindividual and intergroup interactions in mixed‐motive games where both players have equal power, neglecting power differentials that often characterize social interactions in everyday life. We had three key objectives. First, we tested whether the magnitude of the discontinuity effect varies depending on whether the players have equal or unequal power. Second, we compared the behaviour of high‐ and low‐power players, correcting an imbalance in previous research, which has concentrated on high‐power players. Third, we introduced a distinction between unequal‐power stemming from differential control over the other player's outcomes versus differential control over one's own outcomes. Groups were more competitive than individuals and the magnitude of this discontinuity effect did not vary significantly between equal‐ and unequal‐power settings. Further, regardless of whether the interaction was between individuals or groups, unequal (compared to equal) power conduced to competition. Finally, this greater competitiveness in unequal‐power settings was due to the high‐power players. Having high power (compared to equal or low power) increased competition in interindividual and intergroup interactions, irrespective of whether this power derived from greater control over others' or own outcomes.

## POWER EFFECTS ON INTERINDIVIDUAL AND INTERGROUP COMPETITION

**FIGURE 1:**
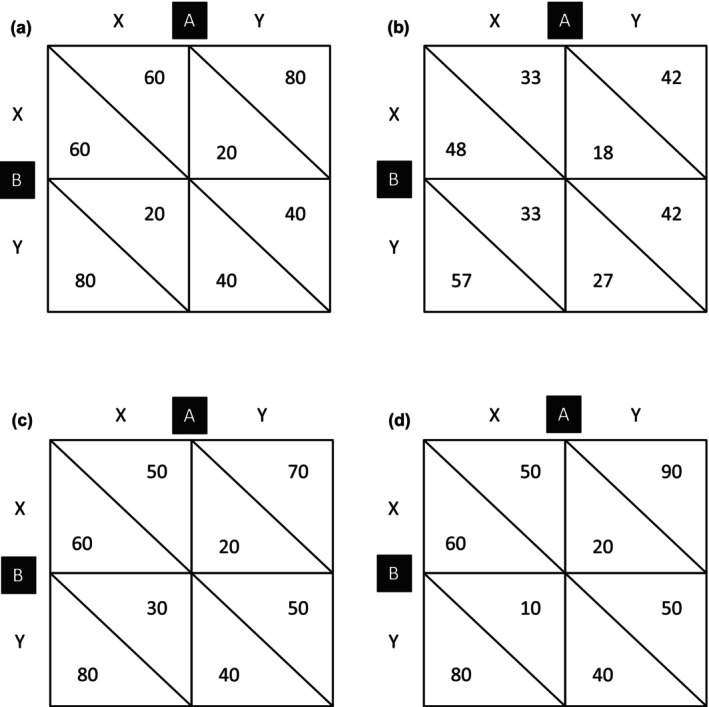
Prisoner's dilemma game (a), unilateral fate control (b), unequal fate control (c), and unequal reflexive control (d) outcome matrices.

Interindividual‐intergroup discontinuity denotes the robust finding that intergroup interactions exhibit more competitive or less cooperative behaviour compared to interactions between individuals (Insko et al., 2001, 2005). Research investigating the differences between interindividual and intergroup interactions has typically done so in mixed‐motive matrix games, such as the prisoner's dilemma game (PDG; Figure 1a). The PDG involves two players (individuals or groups), each choosing between a cooperative (X) and a competitive (Y) option. Outcomes for each player depend on the combination of their choices and can represent rewards or satisfaction levels. Each player maximizes their outcomes by choosing the competitive option, irrespective of the other's choice. Yet, joint selection of that option results in lower outcomes than does mutual cooperation. Succinctly put, “any situation in which you are tempted to do something, but know it would be a great mistake if everybody did the same thing, is likely to be a prisoner's dilemma” (Ridley, 1996, pp. 55–56).

Numerous experiments contrasting interindividual and intergroup interactions within the PDG reveal that intergroup interactions are more competitive (for a meta‐analysis, see Wildschut et al., 2003). John Thibaut labelled this phenomenon a “discontinuity effect” because it highlights the contrast or discontinuity between, on the one hand, the often hostile and aggressive character of intergroup relations and, on the other hand, the otherwise friendly and benign dispositions of the individuals involved. There are various explanations for the discontinuity effect (Wildschut & Insko, 2007). (1) The *fear* explanation posits that intergroup interactions elicit greater distrust than interindividual interactions. In the PDG, if one fears that the other player will act competitively, it is rational to defend oneself by also competing. Other explanations emphasize the role of *greed* in intergroup compared to interindividual interactions. (2) The social‐support explanation suggests that, unlike isolated individuals, group members can garner social support for acting competitively. (3) The identifiability explanation argues that the group context provides anonymity, allowing group members to evade responsibility for competitive choices. (4) The in‐group‐favouring‐norm explanation states that group membership creates normative pressure to benefit the in‐group by competing. (5) The altruistic‐rationalization hypothesis proposes that group members can justify self‐serving competitiveness as being motivated by a concern for the welfare of in‐group members.

Although predominantly studied in mixed‐motive games involving US participants, the discontinuity effect has also been documented in daily diary studies (Pemberton et al., 1996), in experiments using alternative tasks (e.g., economic exchange of folded origami products; Schopler et al., 2001), and among Dutch (Wildschut et al., 2001) and Japanese (Takemura & Yuki, 2007) participants. Notwithstanding such evidence for the effect's generality across settings and populations, researchers have generally investigated interindividual‐intergroup discontinuity in matrix games where both players had equal power, all but neglecting power differentials that so often characterize social interactions in everyday life. Conflicts, whether between individuals or groups, typically involve unequal power. This is especially true in interethnic conflicts, where parties in multiethnic states do not possess equal power and thus experience different levels of threat or security (Rouhana & Fiske, 1995). For instance, both Israeli and Palestinian representatives at the United Nations General Assembly voiced concerns about security threats and proclaimed a desire for peace, but these sentiments were more frequently expressed by the low‐power party (Palestinians) than the high‐power party (Israelis; Ushomirsky et al., 2023). Our primary objective, then, was to address this lacuna by examining whether the discontinuity effect is contingent on whether players (individuals or groups) have equal or unequal power.

## INTERDEPENDENCE THEORY AND TWO TYPES OF POWER

Based on interdependence theory (Kelley & Thibaut, 1978; Thibaut & Kelley, 1959), we operationalized power inequalities by varying the outcomes available to both players in a 2 × 2 matrix game. The theory provides a framework for analysing social relationships via three matrix properties. The first property, fate control (FC), reflects the control a player exerts over the other's outcomes. In symmetrical matrices, like the PDG, FC is identical for both players. For instance, in Figure 1a, the column player's (A) FC is the difference between the row player's (B) average outcomes in the X (70) and Y (30) columns, or 40. The second property, reflexive control (RC), represents the control a player has over their own outcomes. In symmetrical matrices, RC is also the same for both players. In Figure 1a, A's average outcome is 40 in the X column and 60 in the Y column, making their RC the difference between these averages, or 20. The third property, behaviour control (BC), refers to the effect of turn‐taking or alternation of X and Y choices on outcomes. If, by varying their choices, A can induce B to also vary theirs, A has behaviour control over B. In Figure 1a, BC is zero for both players, as their average outcomes are the same across diagonals (50).^1^

Scholars adopting this theoretical approach have conceptualized power in terms of a player's capacity to influence their partner's outcomes or FC: “Specifically, the amount of power A has over B is determined by the range of outcomes through which he potentially could move B” (Schopler, 1965, p. 190). Insko et al. (1983), for example, experimentally manipulated power via rules governing the exchange of origami products, which gave one of three interacting 4‐person groups FC over the other groups. The three groups folded different origami products (e.g., boat, bird) that could be retained or traded and exchanged for money at the end of the study. The groups could earn more money by folding more figures and distributing them optimally between the groups. The high‐power group was advantaged because its products were more valuable and easier to fabricate. Crucially, this group did not have to trade or bargain with the other groups but could simply confiscate their products and then either keep or redistribute them, giving them unilateral FC. The high‐power group earned more money than the other groups and showed “remarkable callousness” (Insko et al., 1983 p. 992) towards them.

In the same tradition, Schopler et al. (1991) manipulated power by varying the outcomes in a 2 × 2 matrix game, giving the high‐power player unilateral FC over the low‐power one. We present their unilateral FC matrix in Figure 1b. In this interaction, A has higher power because their choice affects B's outcomes, whereas B's choice does not affect A's outcomes. To be precise, for A, FC is the difference between B's average outcomes in the X (52.5) and Y (22.5) columns, or 30. For B, FC is the difference between A's average outcomes in the X (37.5) and Y (37.5) rows, or 0. The ability to influence own outcomes, or RC, is the same for both players and thus held constant. That is, by choosing Y (42) instead of X (33), A increases their own outcomes by 9, and so does B. In a 2 × 2 between‐participants design, the researchers varied whether the interaction was between two individuals or two 3‐person groups and whether players had unequal power (unilateral FC) or equal power (PDG). Intergroup interactions were more competitive than interindividual interactions—a discontinuity effect. Further, high‐power players (i.e., the unilateral FC condition) were more competitive than equal‐power players (i.e., the PDG condition). The interaction effect was not significant, supporting the generality of the discontinuity effect across equal‐ and unequal‐power settings. It is important, however, to add two caveats. First, Schopler et al. only analysed the responses of high‐power players and not those of low‐power ones, limiting the scope of their conclusions. Second, few, if any, social relations in everyday life are characterized by purely unilateral FC, whereby the low‐power person or group is incapable of influencing their high‐power partner's outcomes.

Theory and research have emphasized FC as a basis of power. Yet, power can also be defined in terms of a players' ability to control their own outcomes or RC: “… the person having much power is able dependably to insure himself of a wide range of outcomes, from very unfavorable ones (anyone can get these in abundance) to very favorable ones” (Thibaut & Kelley, 1959, p. 89). The two types of power have been referred to as social power (relating to one's control over others' outcomes or FC) and personal power (relating to one's control over own outcomes or RC; Overbeck & Park, 2001). The latter conceptualization of power is evident in the assumption that proposers in the dictator game have more power than those in the ultimatum game (Galinsky et al., 2015; Suleiman, 1996). In the ultimatum game, two players, a proposer and a responder, decide how to split an endowment (e.g., $10). The proposer puts forward a division of the endowment (e.g., $8 for me and $2 for you) and the responder can accept or reject the offer. If the responder rejects the offer, both players receive nothing. In the dictator game, the proposer or ‘dictator’ is given an endowment and decides how to split it. The responder has no other option than to accept what the dictator offers them (if anything). Thus, proposers in the dictator game have complete control over own outcomes whereas those in the ultimatum game do not. Observers view proposers as more powerful in the dictator than ultimatum game, and proposers demand a larger share of the endowment in the dictator than ultimatum game (Sivanathan et al., 2008).

Studies comparing interindividual and intergroup interactions in the ultimatum (Bornstein & Yaniv, 1998; Robert & Carnevale, 1997) and dictator (Luhan et al., 2009) game observed that proposers demanded a larger share of the endowment in intergroup than interindividual interactions, consistent with Schopler et al.'s (1991) finding that the discontinuity effect generalizes to unequal‐power settings (cf. Cason & Mui, 1997). Yet, these studies focused only on the actions of the high‐power player (i.e., the proposer)—a limitation shared with Schopler et al.'s experiment. In addition, the ultimatum and dictator game give the proposer both greater FC and RC than the responder, confounding these two types of power.^2^ Furthermore, these studies did not inform the question of whether the magnitude of the discontinuity effect differs between equal‐ and unequal‐power settings.

## THE PRESENT RESEARCH

Our primary objective was to contrast interindividual and intergroup interactions under circumstances of unequal power and to compare this to the usual research setting in which players have equal power. We operationalized unequal power by creating two outcome matrices (Figure 1c,d). The matrix in Figure 1c manipulates the players' FC, holding constant their RC. By varying their choice, the high‐power player (A) can produce a change of 40 in the low‐power player's (B) outcomes. By contrast, B can only produce a change of 20 in A's outcomes. Both players can produce a change of 20 in their own outcomes. As both players have FC (albeit A more so than B), FC is mutual rather than unilateral, distinguishing this matrix from the one used by Schopler et al. (1991); (Figure 1b). The matrix in Figure 1d manipulates the players' RC, holding constant their FC. The high‐power player (A) can produce a change of 40 in their own outcomes, whereas the low‐power player (B) can only produce a change of 20 in theirs. Both players can produce a change of 40 in the other's outcomes. The matrices allowed us to unconfound FC and RC as sources of power.^3^ Furthermore, the FC difference in the Figure 1c matrix equals the RC difference in the Figure 1d matrix (i.e., 40 vs. 20 in both cases), thereby addressing the criticism that “manipulations of social power often simply involve control over a greater number of valued resources than do manipulations of personal power” (Galinsky et al., 2015, p. 423). The symmetrical PDG matrix in Figure 1a provided the context for equal‐power interactions. The overall level of outcomes or grand mean is the same in all three matrices, and none of them includes an interaction or BC component.

In the equal‐power setting of the PDG, the discontinuity effect is a highly robust phenomenon (Wildschut et al., 2003) and we expected to replicate it. The question was whether the discontinuity effect would be moderated by circumstances of unequal (versus equal) power. In the unequal‐power settings (as in the equal‐power one), competing avoids the lowest possible outcomes in case the other player competes (fear) and achieves the highest possible outcomes in case they cooperate (greed). Explanations positing greater fear and greed in intergroup than interindividual interactions entail, then, that the discontinuity effect should generalize to these settings.

Our second goal was to rectify an imbalance in previous research. Whereas pertinent scholarship has focused predominantly on high‐power actors, we compared high‐ to low‐power players. Unequal‐power settings conduce to more competitive attitudes and behaviours than do equal‐power settings (Schaerer et al., 2018). However, there is a lack of consensus in the literature as to whether, within unequal‐power settings, those with high or low power are generally more competitive. We were guided by a recent study, in which the researchers also operationalized power by varying the outcomes in a 2 × 2 matrix game (du Plessis et al., 2023, Study 2). High‐power individuals reported more trust and, yet, also tended to be more competitive than low‐power ones. These findings suggest that high‐power individuals, more so than low‐power ones, expected the other player to cooperate (i.e., evinced less fear) and were prepared to exploit this vulnerability to maximize their own outcomes (i.e., displayed more greed). Insofar as having high power reduces fear, high‐power groups may not be more competitive than low‐power ones. Yet, to the extent that having high power promotes greed, high‐power groups will be more competitive than low‐power ones (Schopler et al., 1991).

As our final aim, we compared circumstances of unequal control over others' outcomes (i.e., social power) to circumstances of unequal control over own outcomes (i.e., personal power; Overbeck & Park, 2001). Few studies have directly compared social and personal power and, to our knowledge, none have done so by varying systematically control over others' (FC) versus own (RC) outcomes. In the absence of direct evidence, we turned to Cislak et al. (2018), who showed that social power (e.g., “To what extent do you have influence over people in your organization?”) was positively correlated with interpersonal exploitativeness (e.g., “I'm perfectly willing to profit at the expense of others”), whereas personal power (e.g., “I feel I have great control over my life”) was negatively correlated with it. We tentatively hypothesized, then, that circumstances of unequal control over others' outcomes may conduce to more competition than circumstances of unequal control over own outcomes.

We followed Journal Article Reporting Standards. We made data and analysis code available at https://osf.io/m5p8e.

## METHOD

### Participants and design

Three hundred and ninety‐eight University of Southampton undergraduate students (324 women, 74 men) completed the experiment voluntarily (*M*_age_ = 19.43, *SD*_age_ = 2.52). The experiment was approved by the Ethics Committee of University of Southampton (Reference: 19059). We did not preregister the experiment as it was completed prior to the adoption of preregistration as common practice in social psychology.

We randomly assigned participants to conditions in a 2 (interaction type: interindividual vs. intergroup) × 5 (power: equal power vs. high RC vs. low RC vs. high FC vs. low FC). We manipulated interaction type by instructing participants that they would interact with another individual or that they were part of a group that would interact with another group. Instructions did not mention the words “game” or “player,” to avoid framing the interaction as a competition. We manipulated power by varying the payoff structure of the interaction (Figure 1). In the equal‐power condition, participants interacted in the standard, symmetrical PDG (Figure 1a). To operationalize unequal power, we created two asymmetric matrices. One varied the players' FC, holding constant their level of RC (Figure 1c). The other matrix varied the players' RC, holding constant their level of FC (Figure 1d).^4^ We randomly assigned participants to the low‐ or high‐power positions. This created an independent variable with five levels: (1) equal power, (2) low FC, (3) high FC, (4) low RC, (5) high RC. Comparing high‐ and low‐power players created a methodological complexity: when players interact, they influence each other, and the effects of having high versus low power become confounded. We addressed this by letting participants interact on a single trial without communication, yielding independent observations.

We partitioned the 5‐level power variable with four planned orthogonal contrasts (Table 1). Contrast 1 compared the equal‐power condition to the four unequal‐power conditions. Jointly, the next three contrasts are a set of ANOVA contrasts partitioning the variance between the four unequal‐power conditions into a main effect of low versus high power (Contrast 2), a main effect of unequal FC versus unequal RC (Contrast 3), and an interaction effect between low versus high power and unequal FC versus unequal RC (Contrast 4).

**TABLE 1:** Four planned orthogonal contrasts to partition the 5‐level power variable.

	Equal power	Unequal FC		Unequal RC	
		Low power	High power	Low power	High power
Contrast 1	−1	1/4	1/4	1/4	1/4
Contrast 2	0	−1/2	1/2	−1/2	1/2
Contrast 3	0	−1/2	−1/2	1/2	1/2
Contrast 4	0	−1/2	1/2	1/2	‐1/2

A sensitivity power analysis with G*Power 3.1 (Faul et al., 2009) showed that the achieved sample size afforded 80% power to detect effect sizes equal to or greater than *f* = .14 and, in contingency table analyses, *w* = .14 (df = 1, *n* conditions = 10, two‐tailed *α* = .05). This corresponds to a small‐to‐medium effect size.

### Procedure and materials

We instructed participants in the interindividual condition that they would interact with another student, and we told those in the intergroup condition that they were part of a group of students that would interact with another group of students. We informed participants that they would interact with the other player (individual or group) on a single trial. Although we informed participants in the intergroup condition that they were part of a group of around 10 persons,^5^ they completed all dependent measures individually and did not communicate with other members of their group. To create a sense of entitativity or “groupness,” we told them that both groups' choices would be determined following a within‐group majority rule. Each member of their (and the other) group would indicate which choice they want to make, and the experimenter would ostensibly tally these choices to determine each group's final choice. This majority rule creates procedural interdependence among the members within each group, generating perceived entitativity (Insko et al., 2013). Primary studies (Insko et al., 1988; Wildschut et al., 2001) and meta‐analytic findings (Wildschut et al., 2003) indicate that the discontinuity effect is larger when procedural interdependence among group members is present than when it is absent. In fact, the experimenter did not tally the decisions and each participant's choice was treated as an independent unit of analysis.

As in previous research on the discontinuity effect, we gave participants detailed instructions regarding the choice combinations in the payoff matrix, including worked examples of the four possible choice combinations that could occur. Following these instructions, participants completed a brief quiz to test their understanding and had their answers corrected by the experimenter, if necessary. Next, we administered a 9‐item questionnaire assessing participants' expectations concerning the other player (individual or group). Each item used a 7‐point semantic differential scale, anchored with two opposing words or statements (e.g., “honest” vs. “dishonest”; “trustworthy” vs. “untrustworthy”). Scale points were labelled: “definitely,” “probably,” “maybe,” “unsure,” “maybe,” “probably,” “definitely.” We scored the items so that higher scores indicated more negative expectations about the other player and averaged them to create an index (*α* = .86, *M =* 3.43, *SD* = 0.81).

Participants then started the trial. We informed them that the outcomes in the payoff matrix represented credits that they would be able to exchange for a reward at the end of the experiment. The same matrices were used in the interindividual and intergroup conditions. To equate outcomes per participant, those in the intergroup condition were instructed that the outcomes represented the credits that each member of their group could earn. Participants had 1 minute to think about the choice they wanted to make and then to record it by circling “X” (the cooperative choice) or “Y” (the competitive choice). We reminded participants in the intergroup condition that their group's choice would be determined by a majority rule.

Each of the two matrix choices can be selected for a number of different reasons (e.g., the “Y” choice can reflect fear and/or greed).^6^ We, therefore, assessed key reasons or motives identified in the literature to gain insight into the processes that potentially explain choice behaviour. After participants circled their choice, but before being informed of the other player's choice and, in the intergroup condition, their group's majority choice, they completed a validated questionnaire assessing choice reasons (Insko et al., 2013; Pinter et al., 2007; Wildschut et al., 2002). The questionnaire comprised 10 items that were rated on a 7‐point scale (1 = *not at all*, 7 = *very much*). Specifically, the questionnaire assessed five choice reasons with two items each: maximizing own absolute outcomes (MaxOwn; e.g. “I wanted to maximize my [group's] earnings”; *α* = .89, *M =* 4.99, *SD* = 1.44); maximizing own relative outcomes (MaxRel; e.g. “I wanted to earn more than the other person [group]”; *α* = .75, *M =* 4.25, *SD* = 1.55); distrust or fear (e.g. “I did not trust the other person [group]”; *α* = .66, *M =* 3.65, *SD* = 1.43); maximizing the outcomes of both players (MaxJoint; e.g. “I wanted to maximize the joint outcomes of both persons [groups]”; *α* = .94, *M =* 4.17, *SD* = 1.71); and minimizing the difference between the players' outcomes (MinDif; e.g. “I wanted both persons [groups] to earn an equal amount”; *α* = .78, *M =* 3.84, *SD* = 1.64). Choice reasons were significantly (*p* < .001) correlated with competition: MaxOwn, *r* = .51; MaxRel, *r* = .43; fear, *r = *.37; MaxJoint, *r* = −.46; MinDif, *r* = −.45. Finally, participants indicated their gender and age.^7^

## RESULTS

### Negative expectations

We entered the index of negative expectations as a dependent variable in a 2 (interaction type) × 5 (power) ANOVA, with planned contrasts on the 5‐level power variable. We present means and standard deviations in Table 2. Results revealed a significant interaction‐type main effect: negative expectations concerning the other player were higher in the intergroup (*M* = 3.56, *SD* = 0.87) than interindividual (*M* = 3.31, *SD* = 0.72) condition, *F*(1, 385) = 8.79, *p* = .003, *η* ^2^ = .022. The main effect of power was not significant, *F*(4, 385) = 1.22, *p* = .30, *η* ^2^ = .002. The Interaction Type × Power interaction was trending, *F*(4, 385) = 1.95, *p* = .10, *η* ^2^ = .010. We proceeded to partition this omnibus interaction effect with our four planned contrasts.

**TABLE 2:** Means and standard deviations (in parentheses) for negative expectations index as a function of interaction type and power.

	Equal power	Unequal FC		Unequal RC	
		Low power	High power	Low power	High power
Interindividual	3.38 (0.63)	3.44 (0.72)	3.41 (0.81)	3.12 (0.75)	3.20 (0.67)
Intergroup	3.27 (0.78)	3.57 (0.74)	3.71 (0.88)	3.65 (0.97)	3.54 (0.94)

**FIGURE 2:**
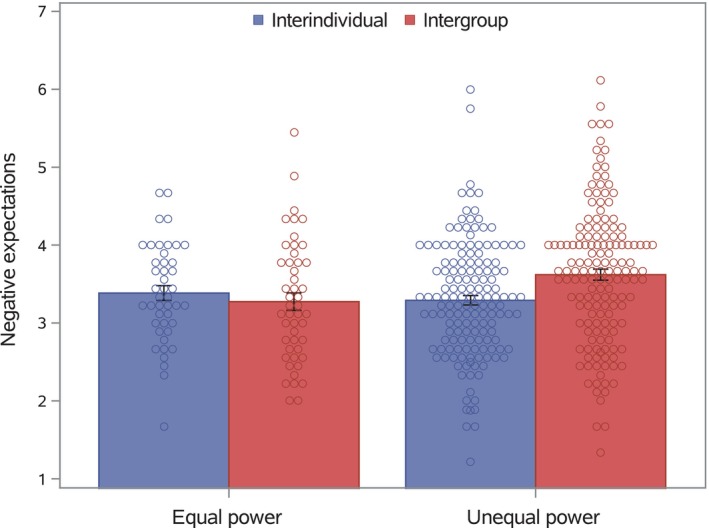
Negative expectations concerning the other player as a function of interaction type and equal power versus unequal power. Error bars represent standard errors.

Tests of planned contrasts revealed a significant interaction effect between interaction type and Contrast 1, the contrast comparing the equal‐power condition to the four unequal‐power conditions, *F*(1, 385) = 5.30, *p* = .022, *η* ^2^ = .014. We display this interaction effect in Figure 2. Tests of simple interaction‐type effects indicated that groups did not have more negative expectations of the other player than individuals in the equal‐power setting, *F*(1, 385) = 0.45, *p* = .50, *η* ^2^ = .001, but did so in the unequal‐power settings, *F*(1, 385) = 12.67, *p* < .001, *η* ^2^ = .031. We also tested the difference between the equal‐ and unequal‐power conditions, separately within the interindividual and intergroup conditions. In the interindividual condition, this difference was not significant, *F*(1, 385) = 0.44, *p* = .51, *η* ^2^ = .001, but it was so in the intergroup condition, *F*(1, 385) = 6.90, *p* = .009, *η* ^2^ = .018. Consistent with evidence for greater distrust or fear in intergroup than interindividual interactions (Wildschut et al., 2004), negative expectations concerning the other player were higher among groups than individuals. This difference was amplified (and significant only) under circumstances of unequal power.

### Competitive choice

We present the proportions of competitive choices in Table 3. Competitive choice was dichotomous (“X” = 0, “Y” = 1) and we entered it as dependent variable in a 2 (interaction type) × 5 (power) generalized linear model, specifying a binomial distribution with a logit link function. Results showed a significant interaction‐type main effect, with competition being higher in the intergroup (*M* = .74, *SD* = .44) than interindividual (*M* = .63, *SD* = .48) condition—a discontinuity effect, χ^2^(1, *N* = 398) = 4.13, *p* = .042. The main effect of power was significant as well, χ^2^(4, *N* = 398) = 19.66, *p* < .001. Below, we partition this omnibus power effect with our four planned contrasts. The Interaction Type × Power interaction was not significant, χ^2^(4, *N* = 398) = 3.70, *p* = .45.

**TABLE 3:** Means and standard deviations (in parentheses) for proportion competition as a function of interaction type and power.

	Equal power	Unequal FC		Unequal RC	
		Low power	High power	Low power	High power
Interindividual	.43 (.50)	.65 (.48)	.84 (.37)	.54 (.50)	.74 (.44)
Intergroup	.69 (.46)	.68 (.47)	.87 (.34)	.74 (.44)	.75 (.43)

*Note*: The standard deviation is calculated as √pq, where *p* is the proportion of competition and *q* is the proportion cooperation (1‐*p*).

Two planned contrasts were significant. A significant Contrast 1 indicated that competition was higher in the unequal‐power condition (*M* = .73, *SD* = .44) than in the pooled equal‐power conditions (*M* = .57, *SD* = .50), *z* = 3.02, *p* = .002. A significant Contrast 2 indicated that, within unequal‐power settings, high‐power players (*M* = .80, *SD* = .40) were more competitive than low‐power ones (*M* = .65, *SD* = .48), *z* = 2.94, *p* = .003.^8^ The two remaining contrasts were not significant, nor were the interaction effects between interaction type and the four contrasts.

Intergroup interactions were more competitive than interactions between individuals. The magnitude of this discontinuity effect did not vary significantly between circumstances of equal versus unequal power, as a function of having high or low power, or between settings with unequal FC versus unequal RC. Further, unequal (compared to equal) power conduced to more competition due to the high‐power individuals and groups, irrespective of whether their power derived from control over others' (FC) or own (RC) outcomes.

### Choice reasons

We present descriptive statistics for choice reasons in Table 4 and ANOVA results in Table 5. Results revealed significant interaction‐type main effects on MaxOwn, MaxRel, and fear. The interaction type effects on MaxJoint and MinDif were trending. Compared to individuals, groups were more concerned with maximizing their own outcomes and with earning more than the other player (i.e., winning). Groups also distrusted the other players more than did individuals. Further, groups tended to be less concerned with maximizing the joint outcomes of both players and minimizing the difference in outcomes between them. The main effect of power was significant for MaxRel only. Below, we partition this omnibus power effect with our four planned contrasts. The Interaction Type × Power interaction effect was not significant for any of the choice reasons.

**TABLE 4:** Means and standard deviations (in parentheses) for choice reasons as a function of interaction type and power.

		Equal power	Unequal FC		Unequal RC	
			Low power	High power	Low power	High power
MaxOwn	Interindividual	4.51 (1.55)	4.91 (1.63)	4.71 (1.33)	4.27 (1.50)	4.84 (1.48)
	Intergroup	5.34 (1.16)	4.96 (1.24)	5.67 (1.19)	5.30 (1.47)	5.39 (1.38)
MaxRel	Interindividual	3.45 (1.54)	3.88 (1.53)	4.12 (1.65)	3.47 (1.46)	4.00 (1.31)
	Intergroup	4.41 (1.58)	4.46 (1.38)	4.92 (1.58)	4.80 (1.47)	5.00 (1.12)
Fear	Interindividual	3.32 (1.55)	3.54 (1.56)	3.53 (1.61)	3.22 (1.52)	3.64 (1.36)
	Intergroup	3.64 (1.43)	3.82 (1.29)	4.22 (1.30)	3.89 (1.12)	3.78 (1.36)
MaxJoint	Interindividual	4.80 (1.59)	4.32 (1.76)	3.95 (1.54)	4.41 (1.80)	4.07 (1.54)
	Intergroup	4.15 (1.90)	4.26 (1.49)	3.67 (1.69)	4.04 (1.85)	3.95 (1.75)
MinDif	Interindividual	4.50 (1.65)	3.77 (1.59)	3.82 (1.63)	3.90 (1.64)	3.92 (1.63)
	Intergroup	3.74 (1.81)	3.84 (1.52)	3.58 (1.78)	3.66 (1.59)	3.63 (1.46)

**TABLE 5:** Inferential statistics from interaction type × power anova on choice reasons.

	Interaction type main effect			Power main effect			Interaction type × power effect		
	*F*(1, 387)	*p*	*η*_p_^2^	*F*(4, 387)	*p*	*η*_p_^2^	*F*(4, 387)	*p*	*η*_p_^2^
MaxOwn	23.70	<.001	.058	1.04	.39	.011	1.54	.19	.016
MaxRel	39.56	<.001	.093	2.44	.046	.025	0.66	.62	.007
Fear	8.69	.003	.022	0.95	.44	.010	0.62	.65	.006
MaxJoint	2.98	.08	.008	1.86	.12	.019	0.39	.81	.004
MinDif	3.10	.08	.008	0.88	.48	.009	0.72	.58	.007

Two planned contrasts were significant for MaxRel. A significant Contrast 1 indicated that concern with winning was higher in the unequal‐power (*M* = 4.33, *SD* = 1.52) than equal‐power (*M* = 3.95, *SD* = 1.63) settings, *F*(1, 387) = 5.24, *p* = .023, *η* ^2^ = .013. A significant Contrast 2 indicated that, within unequal‐power settings, high‐power players (*M* = 4.52, *SD* = 1.49) were more concerned with winning than low‐power ones (*M* = 4.15, *SD* = 1.53), *F*(1, 387) = 4.45, *p* = .035, *η* ^2^ = .011.^9^ The two remaining contrasts were not significant, nor were the interaction effects between interaction type and the four contrasts.

A single choice reason corresponded closely with competitive choices: MaxRel or a concern with winning. Like competition, MaxRel was higher among groups than individuals, in circumstances of unequal than equal power, and among high‐ than low‐power players. This precise match between MaxRel and competition sets the stage for a mediation analysis.

### Mediation of competition by MaxRel

We ran a mediation analysis with SAS PROC CALIS, using maximum likelihood estimation with Satorra‐Bentler adjustment to standard errors (Satorra & Bentler, 1994).^10^ The dependent variable was competition, the mediator was MaxRel, and the independent variables were interaction type and power. Interaction type was contrast coded (interindividual = −1, intergroup = 1) and power was represented in the analysis by the four planned contrasts (Table 1). We did not include the Interaction Type × Power interaction in the model because this effect was not significant for either competition or MaxRel. An indirect effect is statistically significant when its 95% confidence interval (CI) does not include zero. The analysis revealed three significant indirect or mediated effects (denoted as *ab*). First, the effect of interaction type (i.e., the discontinuity effect) on competition was mediated by MaxRel, *ab* = 0.057, 95% CI = [0.035, 0.079], standardized *ab* = .124. Second, MaxRel mediated the effect of unequal (vs. equal) power on competition, *ab* = 0.039, 95% CI = [0.004, 0.074], standardized *ab* = .045. Finally, the effect of having high (vs. low) power on competition was also mediated by MaxRel, *ab* = 0.044, 95% CI = [0.004, 0.083], standardized *ab* = .041. Notwithstanding the inherent limitations of correlational mediation analyses (Rohrer et al., 2022), these results tentatively identify MaxRel or a concern with winning as a key mechanism underlying the observed pattern of competition.

## DISCUSSION

Acknowledging that social interactions in everyday life are characterized by power differentials, our primary objective was to test whether the discontinuity effect would vary between circumstances of unequal versus equal power. Previous studies indicated that the effect generalizes to unequal‐power settings (Bornstein & Yaniv, 1998; Cason & Mui, 1997; Luhan et al., 2009; Robert & Carnevale, 1997; Schopler et al., 1991), yet suffered from a number of limitations, such as focusing exclusively on high‐power players or lacking an equal‐power control condition. We remedied these issues and, additionally, distinguished systematically between unequal‐power stemming from differential control over others' outcomes or FC versus differential control over own outcomes or RC. We replicated the discontinuity effect. Attesting to its generality, the effect did not vary significantly as a function of power distribution (equal vs. unequal), power position (high vs. low), or power type (FC vs. RC). Results further suggested that the discontinuity effect was mediated by MaxRel or concern with winning, consistent with explanations emphasizing the pernicious role of greed in intergroup interactions.

Compared to circumstances of equal power, circumstances of unequal‐power conduce to competitive attitudes and behaviours (Schaerer et al., 2018). Indeed, our results revealed that competition was significantly higher in the unequal‐than‐equal‐power settings, irrespective of whether interactions involved groups or individuals. Interestingly, we found that the mere anticipation of interacting under circumstances of unequal power (compared to equal power) already activated negative expectations regarding the other player, particularly among groups. Still, the literature lacks consensus as to whether, within unequal‐power settings, high‐ or low‐power actors are more competitive. Our second goal was to address this question. In a previous study, high‐ compared to low‐power individuals evinced less fear (i.e., expected the other player to cooperate) but more greed (i.e., competed with the other player; du Plessis et al., 2023, Study 2). We supported the latter but not the former finding. High‐power individuals and groups were significantly more competitive than their low‐power counterparts and this effect was mediated by MaxRel or a concern with winning (i.e., greed). High‐ and low‐power players did not differ significantly on fear. Supplemental tests indicated that low‐power players were not significantly more competitive or concerned with winning than equal‐power ones. The greater competitiveness in unequal‐than‐equal‐power settings, then, was entirely due to greedy high‐power players.

Our final aim was to compare circumstances of unequal control over others' outcomes (i.e., social power) to circumstances of unequal control over own outcomes (i.e., personal power). We operationalized these different types of unequal power with two asymmetric matrices. One varied control over the other player's outcomes or FC, holding constant control over own outcomes or RC (Figure 1c). The other varied RC, holding constant FC (Figure 1d). The difference in FC afforded to the high‐ and low‐power players in the first matrix equalled the difference in RC afforded to them in the second one, rendering the manipulations of social and personal power commensurable—a valuable methodological improvement on past studies (Galinsky et al., 2015). Prior research showed that social power was positively correlated with interpersonal exploitativeness, whereas personal power was negatively associated with it (Cislak et al., 2018). In sharp contrast, our results revealed that high‐power players were more competitive than low‐power ones, regardless of whether their power derived from control over others' or their own outcomes. Evaluating the myriad differences between our work and that of Cislak et al. is beyond the scope of this article. However, a key discrepancy, in our view, is that they measured personal power in general terms that may or may not imply conflict of interest with others (e.g., “I feel I have great control over my life”), whereas we operationalized it specifically in terms of one's capacity to increase own outcomes (RC) under conflict of interest. Given these circumstances, high‐power individuals and groups did not hesitate to exercise RC.

### Null results and statistical power

Whereas the analysis of competitive choice revealed that the omnibus Interaction Type × Power interaction effect was not significant (*p* = .45), inspection of Table 3 shows that the discontinuity effect was numerically larger under conditions of equal than unequal power. Yet, even a focused test of this presumptive interaction effect (i.e., Interaction Type × Contrast 1) yielded a null result (*p* = .15), with a small effect size (*w =* .07). We cannot rule out the possibility that such a small interaction effect exists in the population and we lacked the statistical power to detect it, but raise two points in mitigation. First, with 398 observations, ours is one of the largest experiments on interindividual‐intergroup discontinuity to date. For context, among the 48 experiments included in a meta‐analysis of the discontinuity effect (Wildschut et al., 2003), only one had a larger sample size (Lodewijkx & Rabbie, 1992). Second, it is important to consider the totality of the evidence. The plausibility of a larger discontinuity effect under conditions of equal than unequal power would have been higher had findings for negative expectations and choice reasons aligned with this pattern, but they did not.

## LIMITATIONS AND FUTURE DIRECTIONS

Our work has certain limitations that future investigations can address. To begin with, our sample comprised predominantly female, White British, university‐aged students. An important future direction, therefore, is to examine the generalizability of our findings to other, more diverse populations.

We operationalized power in terms of two matrix properties identified by interdependence theory (Kelley & Thibaut, 1978; Thibaut & Kelley, 1959): FC and RC. Future research could operationalize power in terms of a third property, behaviour control (BC). If, by varying their behaviour, an individual or group can make it desirable for another individual or group to vary their behaviour too, then the former has behaviour control over the latter. In a 2 × 2 outcome matrix, a player's BC over the other player is larger to the extent that this other player's outcome values show a Row × Column interaction pattern. Accordingly, relative power can be manipulated by varying systematically the magnitude of the players' respective interaction patterns.

To create procedural interdependence and the ensuing sense of entitativity (Insko et al., 2013), we told group members that the respective groups' choices would be determined by a majority rule (Insko et al., 2013). Primary studies (Insko et al., 1988; Wildschut et al., 2001) and meta‐analytic findings (Wildschut et al., 2003) indicate that the discontinuity effect is larger when procedural interdependence among group members is present than when it is absent. Thus, the implementation of a majority rule can be viewed as creating conditions under which the discontinuity effect can be observed, that is, as strengthening the interaction‐type manipulation. By so doing, however, we created a specific type of laboratory group. Future research on the moderating role of power in individual‐intergroup discontinuity would do well to study other types of groups (e.g., groups with face‐to‐face interaction within and between groups; naturalistic groups).

Future research could also fruitfully consider the role of individual differences. Examining the role of self‐views in dispute resolution, Howard et al. (2007) showed that a more interdependent self‐construal (i.e., a self‐view that emphasizes connectedness to others), compared to a more independent self‐construal (i.e., a self‐view that emphasizes uniqueness from others), led individuals to use their power more benevolently in interindividual negotiations. Yet, paradoxically, a more interdependent (compared to independent) self‐construal led group members to use their power more exploitatively in intergroup negotiations. Howard et al.'s conclusion that their work “calls into question the view that a concern for others will consistently lead people to be more trustworthy as power holders” (p. 629) resonates with findings concerning the divergent implications of guilt proneness, a personality trait also characterized by concern for others (Tangney, 2003), in interindividual versus intergroup interactions (Wildschut & Insko, 2006). Whereas guilt proneness is associated with decreased interindividual competition, it is associated with the increased intergroup competition when members' concern for the in‐group is rendered salient (Cohen et al., 2006; Pinter et al., 2007). Thus, high concern for others' welfare, though often desirable, may aggravate rather than constrain the abuse of power in intergroup relations. Hobbes (1983) captured the essence of this quandary when he wrote that “Force, and Fraud, are in warre the two Cardinall vertues” (p. 66).

We identified MaxRel or a concern with winning as a potential mediating mechanism underlying the observed pattern of competition. Evidence for mediation is suggestive rather than conclusive, however, because MaxRel and competition were both measured and, hence, associations between them are correlational and do not allow strong causal inferences (Rohrer et al., 2022). Nonetheless, these analyses can be informative because they place the mediation hypothesis at risk (Fiedler et al., 2011). Future research could harness alternative strategies for testing mediation, as implemented by experimental‐causal‐chain and moderation‐of‐process designs (Spencer et al., 2005).

Finally, participants interacted on a single trial only. It is important, in future research, to assess the generalizability of our findings to interactions that extend over multiple trials (Insko et al., 2001). In our study, the column player always achieved higher outcomes by competing, no matter what choice the row player made. In terms of immediate outcome maximization, then, the competitive choice is optimal, which incentivizes the use of power. Over many trials, however, the situation changes. If both players always pursue their immediate self‐interest, they achieve the collectively suboptimal outcomes associated with mutual competition. To obtain better collective outcomes in the long run, the players should move from competing to cooperating, which may curb the use of power. Related to this, power is constrained to the extent that its use incurs penalties. Over many trials, the low‐power player can wield their control over the high‐power player's outcomes (i.e., their counterpower), small as it might be, to punish the use of power. By so doing, the low‐power player can limit the high‐power player's “usable power” or “the power that it is convenient and practicable … to use” (Thibaut & Kelley, 1959, p. 107).

In real‐world settings, low‐power actors' repeated application of counterpower can promote in high‐power actors three perceptions that connect long‐term thinking with mutual cooperation: “(a) perceived dependence on the other (i.e., a recognition of the importance of the other's cooperation); (b) pessimism about the likelihood that the other can be exploited (i.e., that he will cooperate unilaterally for any period of time); and (c) insight into the necessity of cooperating with the other in order to achieve his cooperation” (Pruitt & Kimmel, 1977, p. 375). Alternative, moral constraints on high‐power actors' usable power, including norms of fairness and equality, are more salient in interindividual than intergroup relations (Wildschut & Insko, 2006), making the judicious use of counterpower especially important in the latter context.

In conclusion, aiming to correct past neglect of power differentials that typically characterize social interactions in everyday life, we uncovered three key findings. (1) We replicated the discontinuity effect. Supporting its generality, the effect did not vary significantly between circumstances of equal and unequal power. (2) Competition was higher under circumstances of unequal than equal power. (3) High‐power players were responsible for the greater competitiveness in unequal‐than‐equal‐power settings. A single mechanism, MaxRel or concern with winning, may underlie all three findings, making it a prime target for real‐world interventions to mitigate the (ab)use of power. Such interventions are likely to be more effective when they highlight the low‐power actor's counterpower and associated long‐term benefits of mutual cooperation.

## AUTHOR CONTRIBUTIONS

**Tim Wildschut:** Conceptualization; investigation; writing – original draft; methodology; visualization; formal analysis; data curation. **Chester A. Insko:** Conceptualization; writing – review and editing; methodology.

## CONFLICT OF INTEREST STATEMENT

The authors declare no conflicts of interest.

## Data Availability

We made data and analysis code available at https://osf.io/m5p8e.

## Footnotes

^1^
A player's BC over the other player is larger to the extent that this other player's outcome values show a statistical interaction pattern (i.e., a difference between the average outcomes across diagonals; Thibaut & Kelley, 1959, pp. 103–104). To illustrate this, in Figure 1a, B's average outcome in top‐left (60) to bottom‐right (40) diagonal is 50. This player's average outcome in the top‐right (20) and bottom‐left (80) diagonal is also 50. The difference between the diagonals is 0. Thus, there is no statistical interaction pattern, and A has no BC over B (or vice versa, given the matrix symmetry).

^2^
Assuming that the responder in the ultimatum game prefers any positive payoff greater than zero (i.e., the standard game‐theoretical postulate of outcome maximization), and the proposer knows this, the proposer will offer them only the minimal amount, and the responder will accept (Kugler et al., 2012). Participants' actual responses do not support this prediction. In the ultimatum game, proposers tend to offer just under half of the endowment, and responders often reject offers they see as unfair (Camerer & Thaler, 1995).

^3^
We constructed the unequal‐power matrices so that the RC:FC ratio equalled −0.5 for the high‐power player and − 1.0 for the low‐power player in both matrices. In the symmetrical PDG, the ratio equalled −0.5 for both players.

^4^
We created two versions of each unequal‐power matrix, so that participants were always the column player, A, regardless of whether they were randomly assigned to the high‐ or low‐power position.

^5^
Undergraduate students at University of Southampton are assigned to a “tutor group” of around 10 students. We told participants in the intergroup condition that their tutor group would interact with another tutor group.

^6^
Because the “Y” choice need not reflect a concern with winning, it is also referred to as the non‐cooperative choice or defection (for a discussion of labelling conventions, see Pruitt & Kimmel, 1977, p. 372). We referred to it as the competitive choice because, in everyday parlance, competition is a common antonym of cooperation.

^7^
The first questionnaire participants completed, which preceded the assessment of negative expectations, was a measure of dispositional paranoia, the 20‐item Paranoia Scale (Fenigstein & Vanable, 1992). We included this scale for purposes unrelated to the present experiment and did not analyse the data.

^8^
The significant difference, within unequal‐power settings, between high‐ and low‐power players raises the question of whether low‐power players were more competitive than equal‐power ones. A post‐hoc contrast between these conditions indicated that they did not differ significantly, *z* = 1.35, *p* = .18.

^9^
The significant difference, within unequal‐power settings, between high‐ and low‐power players raises the question of whether MaxRel was higher among low‐ than equal‐power players. A post‐hoc contrast between these conditions indicated that they did not differ significantly, *F*(1, 387) = 1.32, *p* = .25, *η* ^2^ = .003.

^10^
This method generates parameter estimates that are unbiased asymptotically and adjusts for non‐normal data so that obtained standard errors are accurate.
